# Comparison of tocilizumab as monotherapy or with add-on disease-modifying antirheumatic drugs in patients with rheumatoid arthritis and inadequate responses to previous treatments: an open-label study close to clinical practice

**DOI:** 10.1007/s10067-014-2857-y

**Published:** 2015-01-22

**Authors:** Vivian P. Bykerk, Andrew J. K. Östör, José Alvaro-Gracia, Karel Pavelka, José Andrés Román Ivorra, Winfried Graninger, William Bensen, Michael T. Nurmohamed, Andreas Krause, Corrado Bernasconi, Maher Aassi, Jean Sibilia

**Affiliations:** 1 Inflammatory Arthritis Center, Hospital for Special Surgery, 535 East 70th Street, New York, NY 10021 USA; 2 Department of Rheumatology, Mount Sinai Hospital, Toronto, ON Canada; 3 University of Cambridge, Cambridge, UK; 4 Hospital Universitario de la Princesa, IIS Princesa, Madrid, Spain; 5 Institute of Rheumatology, Prague, Czech Republic; 6 Hospital Universitario La Fe, Valencia, Spain; 7 Medical University of Graz, Graz, Austria; 8 St Joseph’s Hospital/McMaster University, Hamilton, ON Canada; 9 VU University Medical Center, Amsterdam, Netherlands; 10 Immanuel Hospital, Berlin, Germany; 11 F. Hoffmann-La Roche, Basel, Switzerland; 12 CHU Hautepierre, Strasbourg, France

**Keywords:** Disease activity, DMARDs (biologic), DMARDs (synthetic), Monotherapy, Open-label, Rheumatoid arthritis, Tocilizumab, Tumor necrosis factor-α inhibitor

## Abstract

This was an exploratory analysis comparing the safety and efficacy of tocilizumab monotherapy with those of tocilizumab in combination with disease-modifying anti-rheumatic drugs (DMARDs). Data were from a single-arm, nonrandomized, open-label, 24-week study in patients with rheumatoid arthritis in which patients with inadequate responses to DMARDs or tumor necrosis factor-α inhibitors received tocilizumab 8 mg/kg intravenously every 4 weeks plus methotrexate/other DMARD(s) combination therapy. If they were intolerant of methotrexate/other DMARD, patients received tocilizumab monotherapy. Effectiveness endpoints included American College of Rheumatology (ACR) responses (ACR20/50/70/90) and disease activity score using 28 joints (DAS28). Of 1,681 patients, 239 received tocilizumab monotherapy, and 1,442 received combination therapy. Methotrexate was the most common DMARD (79 %) used in combination therapy. The frequency of adverse events (AEs), serious AEs, and AEs leading to withdrawal were similar between tocilizumab monotherapy (82.4, 7.9, and 5.4 %, respectively) and combination therapy (76.6, 7.8, and 5.1 %, respectively). No differences in ACR20/50/70/90 responses were observed between treatment groups (66.9 %/43.5 %/23.8 %/10.0 % vs 66.9 %/47.2 %/26.8 %/8.5 %, respectively; *p* > 0.12 for all individual comparisons, including ACR50 propensity score analyses). The decrease in DAS28 was also similar between treatment groups (mean ± standard deviation: −3.41 ± 1.49 for tocilizumab monotherapy vs −3.43 ± 1.43 for combination therapy; *p* > 0.33 all analyses, including propensity score analyses). Tocilizumab had a comparable safety profile, and was similarly effective, when used as monotherapy or in combination with DMARDs in a broad population of patients with rheumatoid arthritis.

**Electronic supplementary material:**

The online version of this article (doi:10.1007/s10067-014-2857-y) contains supplementary material, which is available to authorized users.

## Introduction

The current recommendation for patients with rheumatoid arthritis (RA) not responding adequately to methotrexate (MTX) or other disease-modifying antirheumatic drugs (DMARDs) is to coadminister a biologic [1]. However, approximately one-third of RA patients treated with biologics receive them as monotherapy (without DMARDs) [2]. Patients may discontinue DMARDs because of toxicity [3], contraindications, or personal choice [4].

The safety and efficacy of tocilizumab have been demonstrated in RA patients who have inadequate responses to MTX/DMARDs (MTX/DMARD-IR) or tumor necrosis factor-α inhibitor (TNFi) agents (TNFi-IR) [5]. Tocilizumab monotherapy has been demonstrated to be more effective than MTX [6, 7] or DMARD [8] monotherapy. Further, tocilizumab combined with MTX has not been shown to be superior to tocilizumab monotherapy in MTX-IR patients [9]. In ACT-SURE, a previously reported nonrandomized, open-label study, tocilizumab was administered under conditions more typical of clinical practice [10]; investigators could administer tocilizumab as monotherapy or combined with DMARDs at their discretion if the patient was able to tolerate DMARDs. Here we describe and compare these two treatment approaches.

## Methods

### Study design

In this 24-week, phase 3b, single-arm, nonrandomized, open-label safety and effectiveness study conducted July 2008 through March 2010, patients were enrolled from 264 centers in 25 countries. Patients were ≥18 years of age, had moderate to severe active RA for ≥6 months, and had inadequate clinical responses to ≥1 DMARD and/or TNFi therapy. Tocilizumab 8 mg/kg was administered intravenously every 4 weeks. Patients receiving a TNFi agent before baseline (as monotherapy or combined with nonbiologic DMARDs) replaced it with tocilizumab; patients receiving DMARDs alone added tocilizumab to their regimens; and patients receiving a TNFi agent alone discontinued it and initiated tocilizumab monotherapy. DMARDs were maintained at stable doses (choice and dose at investigator discretion); if DMARDs were poorly tolerated (as determined by the investigator), tocilizumab was administered as monotherapy without a washout period. The DMARD dosage was modified (reduced) only for safety reasons. Oral corticosteroids (≤10 mg/day prednisone or equivalent) and nonsteroidal anti-inflammatory drugs (NSAIDs) had to be used at stable doses for at least 25 of 28 days before treatment (day 1). Oral corticosteroids were to be kept constant throughout the study unless tapering was required for safety reasons. If MTX-related adverse events (AEs) or laboratory abnormalities developed, dose reduction or change in route was considered for the patient before study withdrawal. Ethical and regulatory approval and patients’ written informed consent were obtained in accordance with the Declaration of Helsinki; Good Clinical Practice was followed.

### Assessments

Safety endpoints included AEs, serious AEs (SAEs), serious infections, neutrophil counts, and liver transaminase levels*.* Effectiveness endpoints included ACR20/50/70/90 responses, European League Against Rheumatism (EULAR) responses, DAS28 using erythrocyte sedimentation rate, and simplified and clinical disease activity index (SDAI and CDAI, respectively). Health Assessment Questionnaire–Disability Index (HAQ-DI) data were also collected. For individual components, missing data were imputed using last-observation-carried-forward until withdrawal for joint counts only; for ACR and EULAR responses, missing data were considered no response. Safety and effectiveness were assessed monthly.

### Statistical analyses

In this exploratory analysis, which addresses prespecified protocol-defined study objectives, patients were grouped according to initial treatment: tocilizumab monotherapy (monotherapy group) or tocilizumab plus ≥1 DMARD (combination group). Descriptive statistics were used for incidences of AEs and SAEs, and two-sided Clopper–Pearson 95 % confidence intervals were calculated unless specified otherwise. To test the “tocilizumab monotherapy = combination” hypothesis for effectiveness in this nonrandomized setting, logistic or analysis of covariance (ANCOVA) models adjusted for previous treatment (DMARD-IR/TNFi-IR [TNFi previous use: >2 months before baseline vs TNFi recent use: ≤2 months before baseline], known to have different efficacy outcomes [10]) were used with baseline DAS28, CDAI, or SDAI, as applicable, as relevant confounders. For two key endpoints, ACR50 response and DAS28 change, supportive post hoc analyses used propensity scores [11] computed using a logistic regression model (Electronic supplementary material (ESM) Table S1). Five matched groups were created based on quintiles of the score. Overall DAS28 difference and Cochran–Mantel–Haenszel statistics for ACR50 response were computed; propensity score was included as a covariate in multivariate models.

## Results

### Patients

The safety and intent-to-treat (ITT) populations included 1,681 patients (239 [14 %], monotherapy; 1,442 [86 %], combination therapy; ESM Fig. S1). Overall, patients had established RA (mean duration, 9.6 years) with high disease activity (mean DAS28, 6.0) and were highly treatment experienced (mean number of previous nonbiologic DMARDs [not including current treatment], 1.3; 42 % had used TNFi agents [mean, 1.4]; Table 1). Disease duration and many baseline disease activity measures were higher in the monotherapy group, consistent with the fact that the majority (72 %) of monotherapy patients were TNFi-IR. In the combination group, MTX was the most common DMARD (79 %); 3 % of monotherapy patients started a DMARD (all MTX) during the study.

**Table 1:** Baseline demographics and characteristics

	Tocilizumab monotherapy *n* = 239	Tocilizumab + DMARD (s) *n* = 1,442	All patients *N* = 1,681	*p* ^a^
Female, %	82	81	81	0.79 (F)
Age, years	55.2 (12.3)	53.2 (12.3)	53.5 (12.3)	0.034 (W)
Duration of RA, years	11.0 (9.7)	9.4 (8.6)	9.6 (8.8)	0.0050 (W)
DAS28	6.2 (1.3)	5.9 (1.2)	6.0 (1.2)	0.0010 (W)
SJC	14.0 (10.7)	12.6 (8.9)	12.8 (9.2)	0.22 (W)
TJC	25.5 (15.9)	22.4 (14.8)	22.8 (15.0)	0.0030 (W)
Patient global VAS	66.6 (20.7)	61.8 (21.2)	62.5 (21.2)	0.0015 (W)
Physician global VAS	62.4 (18.4)	58.2 (17.7)	58.8 (17.9)	0.0008 (W)
Patient pain VAS	61.3 (22.5)	56.9 (22.6)	57.5 (22.6)	0.0047 (W)
CRP, mg/dL	2.5 (2.8)	1.8 (2.8)	1.9 (2.8)	<0.0001 (W)
ESR, mm/h	44.6 (28.5)	38.3 (26.5)	39.2 (26.8)	0.0010 (W)
HAQ-DI	1.7 (0.6)	1.5 (0.6)	1.5 (0.6)	<0.0001 (W)
CDAI	36.5 (15.0)	34.0 (13.4)	34.3 (13.6)	0.019 (W)
SDAI	38.9 (16.1)	35.8 (14.2)	36.3 (14.5)	0.0047 (W)
DMARD-IR, *n* (%)	66 (28)	910 (63)	976 (58)	<0.0001 (C)
TNFi previous use,^b^ *n* (%)	62 (26)	236 (16)	298 (18)	
TNFi recent use,^c^ *n* (%)	111 (46)	296 (21)	407 (24)	
Baseline DMARD use, %				
MTX	0	79	67	
Hydroxychloroquine	0	16	14	
Sulfasalazine	0	13	11	
Leflunomide	0	13	11	
Baseline oral corticosteroid use, *n* (%)	124 (51.9)	733 (50.8)	857 (51.0)	
Baseline oral corticosteroid dose, mg/d^d^	7.8 (3.6)	7.1 (3.5)	7.2 (3.5)	
Baseline DMARD dose				
MTX, mg/week	0	17.5 (7.3)	17.5 (7.3)	
Hydroxychloroquine, mg/day	0	331.8 (151.0)	331.8 (151.0)	
Sulfasalazine, g/day	0	1.9 (0.6)	1.9 (0.6)	
Leflunomide, mg/day	0	18.4 (4.6)	18.4 (4.6)	

Data are presented as mean (*SD*) unless stated otherwise

*VAS* Visual Analogue Scale

^a^Between-group comparisons: *F* Fisher exact test; *W* Wilcoxon rank sum test; *C* chi-square test for no association

^b^Patients who did not use TNFi for >2 months before baseline

^c^Patients who used TNFi for ≤2 months before baseline

^d^Dose in prednisone equivalents, considering only patients receiving corticosteroids

### Safety

The frequencies of AEs (82.4 vs 76.6 % of patients in the monotherapy and combination groups) and AEs leading to withdrawal (5.4 vs 5.1 %) were similar between treatment groups (Table 2). The incidences of SAEs (19.4 vs 20.2/100 patient-years in monotherapy and combination therapy groups) and serious infections (4.6 vs 5.2/100 patient-years), which were the most common SAE, were also similar. Grade 3/4 neutropenia and transaminase elevations occurred less frequently with monotherapy than with combination therapy (treatment modifications after laboratory events were made according to the tocilizumab label). Three of four reported deaths occurred in the combination therapy group (Table 2 [10]).

**Table 2:** Safety outcomes

	Tocilizumab monotherapy *n* = 239	Tocilizumab + DMARD (s) *n* = 1,442	All patients *N* = 1,681
Exposure, 100 PY	1.08	6.59	7.67
AEs, % (95 % CI)^a^	82.4 (77.0, 87.0)	76.6 (74.3, 78.7)	77.4 (75.3, 79.4)
SAEs, % (95 % CI)^a^	7.9 (4.9, 12.1)	7.8 (6.4, 9.3)	7.8 (6.6, 9.2)
SAEs, rate/100 PY (95 % CI)^b^	19.4 (12.0, 29.7)	20.2 (16.9, 23.9)	20.1 (17.0, 23.5)
AEs leading to withdrawal, % (95 % CI)^a^	5.4 (2.9, 9.1)	5.1 (4.0, 6.3)	5.1 (4.1, 6.3)
Infections, % (95 % CI)^a^	38.1 (31.9, 44.6)	34.9 (32.4, 37.4)	35.3 (33.0, 37.7)
Serious infections, % (95 % CI)^a^	2.1 (0.7, 4.8)	2.1 (1.5, 3.0)	2.1 (1.5, 3.0)
Serious infections, rate/100 PY (95 % CI)^b^	4.6 (1.5, 10.8)	5.2 (3.6, 7.2)	5.1 (3.6, 6.9)
Deaths, *n*	1^e^	3^f^	4
Grade 3/4 neutropenia^c^ at ≥1 time point, %	1.7	3.3	3.1
ALT shift from normal at baseline to >1.5–3× ULN at any time,^d^ % (*n*)	9.2 (22)	12.9 (186)	12.4 (208)
ALT shift from normal at baseline to >3× ULN at any time,^d^ % (*n*)	1.7 (4)	2.1 (31)	2.1 (35)
AST shift from normal at baseline to >1.5–3× ULN at any time,^d^ % (*n*)	2.9 (7)	5.2 (75)	4.9 (82)
AST shift from normal at baseline to >3× ULN at any time,^d^ % (*n*)	0 (0)	0.7 (10)	0.6 (10)

*ALT* alanine aminotransferase, *AST* aspartate aminotransferase, *CI* confidence interval, *PY* patient-year, *ULN* upper limit of normal

^a^Two-sided 95 % Clopper-Pearson CI

^b^Two-sided 95 % Poisson CI

^c^Only one case of grade 4 neutropenia was reported in the study (tocilizumab + DMARD[s] group)

^d^Highest postbaseline value

^e^Streptococcal sepsis, considered possibly related to study medication

^f^Aortic dissection, considered unrelated to study medication; cardiac arrest (*n* = 2; 1 considered possibly related to study medication)

### Effectiveness

**Fig. 1:**
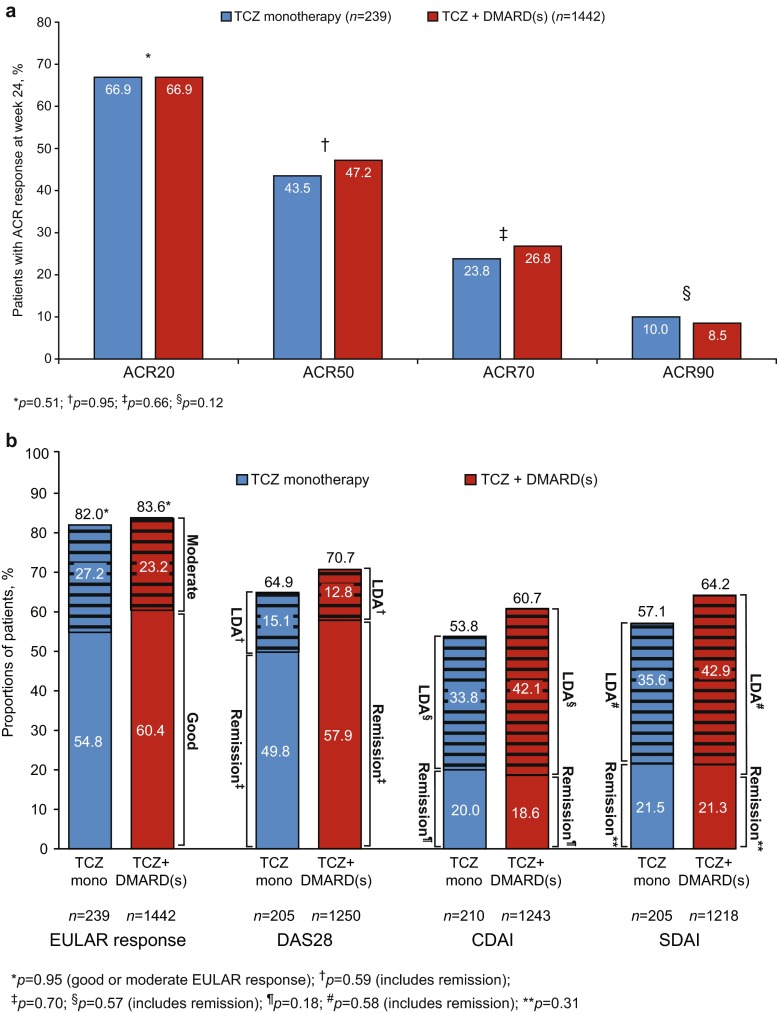

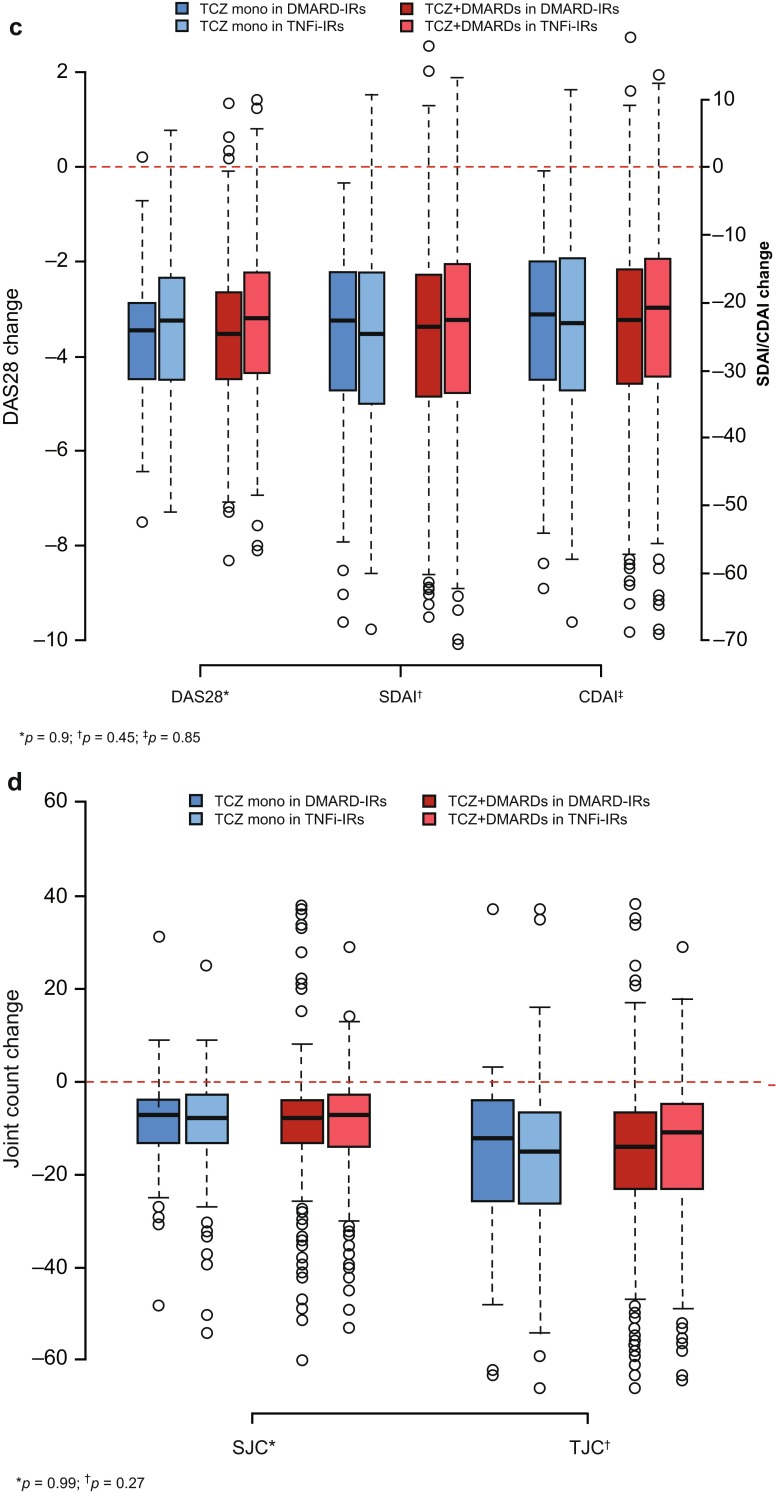
Effectiveness at week 24. **a** ACR20/50/70/90 responses. **b** DAS28, CDAI, and SDAI responses. **c** Change from baseline in DAS28, CDAI, and SDAI. **d** Change from baseline in swollen joint count (*SJC*) and tender joint count (*TJC*). **a** *p* values were calculated by logistic regression analysis adjusted for previous treatment (DMARD-IR/TNFi-IR [previous TNFi use/recent TNFi use]) and baseline DAS28. Nonresponder imputation was performed for patients who withdrew or for whom responses were missing. **b** *Hatched lines* represent moderate EULAR response or low disease activity. EULAR good response: DAS28 ≤ 3.2 at week 24 and change of >−1.2. EULAR moderate response: DAS28 ≤ 3.2 at week 24 and change of <−0.6 to ≥−1.2 or <−1.2; DAS28 > 3.2 to ≤5.1 at week 24 and change of <1.2. DAS28: low disease activity (LDA), ≥2.6 to 3.2; remission, <2.6. CDAI: LDA, >2.8 to ≤10; remission, ≤2.8. SDAI: LDA, >3.3 to ≤11; remission, ≤3.3. *p* values calculated by logistic regression analysis adjusted for previous treatment (DMARD-IR/TNFi-IR [previous TNFi use/recent TNFi use]) and baseline DAS28, CDAI, or SDAI, as applicable. **c** TCZ monotherapy in DMARD-IR patients, *n* = 66; TCZ monotherapy in TNFi-IR patients, *n* = 173; TCZ + DMARDs in DMARD-IR patients, *n* = 910; TCZ + DMARDs in TNFi-IR patients, *n* = 532. *p* values were calculated by Wilcoxon rank-sum test and compare TCZ monotherapy and TCZ combination therapy (disregarding the DMARD-IR-TNFi-IR split). **d** TCZ monotherapy in DMARD-IR patients, *n* = 66; TCZ monotherapy in TNFi-IR patients, *n* = 173; TCZ + DMARDs in DMARD-IR patients, *n* = 910; TCZ + DMARDs in TNFi-IR patients, *n* = 532; *p* values were calculated by Wilcoxon rank-sum test and compare TCZ monotherapy and TCZ combination therapy (disregarding the DMARD-IR-TNFi-IR split)

Eighty-seven percent of the ITT population completed the study (complete data for DAS28 and ACR core set (ESM Table S2) were available for 87 and 83 % of patients, respectively). Percentages of patients achieving ACR20/50/70/90 responses at week 24 were 66.9, 46.6, 26.4, and 8.7 %, respectively, and were similar between treatment groups (maximum difference, 3.7 %; *p* > 0.12, all comparisons, including an analysis of ACR50 using propensity scores; Fig. 1a, ESM Table S1). ACR20/50/70 responses occurred as early as week 4 and improved through week 24 (ESM Fig. S2).

At week 24, no statistically significant difference was found between treatment groups in EULAR good/moderate responses, DAS28 < 2.6 (remission), or DAS28 low disease activity (LDA; Fig. 1b). EULAR good/moderate responses and DAS28 < 2.6 were achieved as early as week 4, and percentages of patients achieving EULAR good/moderate responses were maintained through week 24 (ESM Figs. S3A, S3B). Percentages with DAS28 < 2.6 increased through weeks 20 and 24 in the monotherapy and combination groups, respectively (ESM Fig. S3B); overall, DAS28 < 2.6 was achieved in 56.8 % of patients. At week 24, numerically higher proportions of combination therapy patients than monotherapy patients achieved CDAI and SDAI LDA; CDAI and SDAI remission rates were similar (Fig. 1b), as were proportions of patients achieving ACR/EULAR Boolean remission (11.3 % monotherapy vs 11.9 % combination; *p* = 0.54). Decreases from baseline in DAS28 (mean ± standard deviation, −3.41 ± 1.49 monotherapy vs −3.43 ± 1.43 combination), CDAI, and SDAI were also similar between treatment groups, with nonsignificant differences in univariate (Wilcoxon rank-sum test; Fig. 1c) or multivariate (ANCOVA) analyses, including analysis of DAS28 change using propensity scores (*p* > 0.33 for all analyses; ESM Table S1). Differences in effectiveness between treatment groups were small and nonsignificant in the subgroups of patients with and without previous TNFi therapy (Fig. 1c and d, ESM Table S3).

Decreases in joint counts from baseline to week 24 were similar between treatment groups (Fig. 1d). At week 24, decreases in HAQ-DI were comparable between groups (difference, 0.03; *p* = 0.48), but a higher percentage of combination therapy patients (73.4 %) than monotherapy patients (68.4 %) achieved clinically meaningful improvement in HAQ-DI (*p* = 0.030; ESM Fig. S4). Effectiveness outcomes in patients in the combination therapy group who received methotrexate (ESM Table S4) and in the monotherapy group were similar.

In a subset of study centers, patients who experienced at least moderate EULAR response after 24 weeks of treatment were eligible to enter a long-term extension study. Mean DAS28, DAS28 < 2.6 responders, ACR20/50/70/90 responders, and percentages of patients who achieved clinically meaningful improvement in HAQ-DI were similar between treatment groups after 6 months of the extension study (ESM Table S5).

No evidence for an interaction between baseline DAS28 and treatment type was found using models for DAS28 and ACR20, ACR50, and EULAR responses at week 24 with additional adjustment for previous treatment. This indicates that baseline disease activity is not a valuable response predictor for guiding a choice between monotherapy and combination therapy.

## Discussion

In contrast to most phase 3 studies, ACT-SURE evaluated a population treated closer to actual clinical practice. Patients were from tertiary academic centers, nonacademic centers, and private practices with virtually no restrictions on previous DMARD and TNFi types, dosages, and combinations. Patients could be treated with a variety of concomitant medications including add-on DMARDs, NSAIDs, and corticosteroids. Our focus was on the comparison between TCZ used as monotherapy or in combination with nonbiologic DMARDs.

Relevant limitations of this study were the open-label design, which potentially introduced bias because clinical assessments were not performed in a blinded manner, the absence of randomization, the focus on short-term safety and clinical outcomes, and the discontinuation of DMARDs at study entry if poorly tolerated, at the discretion of the rheumatologist. Monotherapy patients had more severe disease at entry, reflecting the fact that this population consisted primarily of TNFi-IR patients. Reports indicate that approximately one-third of RA patients using biologics receive them as monotherapy [2]. This observation is in line with the rate of tocilizumab monotherapy in TNF-IR patients (25 %); however, only 7 % of DMARD-IR patients received tocilizumab monotherapy as their first biologic. Additional factors such as lack of efficacy and poor adherence may also contribute to DMARD discontinuation over time during biologic treatment [12].

Slightly higher incidences of transaminase elevations and neutropenia with monotherapy were observed but were not associated with severe clinical consequences. Overall the safety profiles in patients receiving tocilizumab monotherapy and combination therapy overlapped, consistent with results of a recent double-blind study [9]. A larger study would be needed for an appropriate comparison of rates of uncommon events such as serious infections.

This exploratory analysis did not indicate a sizable difference in clinical effectiveness between tocilizumab monotherapy and combination therapy. To account for potential confounders, including previous treatments, multivariate analysis and propensity scores were used. Multivariate analysis confirmed previous reports from ACT-SURE suggesting that TNFi-IR patients represent a difficult-to-treat population in whom tocilizumab is slightly less effective than it is in TNF-naive patients [10]. However, in both TNFi-IR and DMARD-IR subpopulations, the effectiveness of monotherapy was comparable to that of combination therapy at week 24. Retrospectively, this study would have had at least 80 % power to detect a difference of 0.3 in DAS28 change and a 9.5 % ACR50 response difference. Thus, differences in effectiveness generally considered clinically relevant would likely have been identified.

These data are consistent with findings in other tocilizumab monotherapy trials. In MTX-naive or 6-month MTX-free patients and in MTX-IR patients, tocilizumab monotherapy resulted in higher ACR responses than MTX monotherapy [6, 8]. The efficacy of tocilizumab monotherapy was also demonstrated in a double-blind, head-to-head trial against adalimumab monotherapy in patients for whom MTX was considered inappropriate (because of lack of efficacy or intolerance) [13]. In most clinical trials, efficacy did not differ substantially between tocilizumab monotherapy and combination therapy at 6 months, whereas long-term comparative data are lacking. Further studies are needed to confirm the sustained response to tocilizumab monotherapy in RA patients who experienced inadequate response to previous treatments. In the 24-week analysis of a randomized study in 556 MTX-IR patients [9], no clinically relevant superiority was demonstrated with an MTX plus tocilizumab add-on strategy over switching to tocilizumab monotherapy. Small numerical differences in the primary and some secondary endpoints favoring combination therapy were not considered clinically meaningful. Similarly, in an open-label, randomized study in DMARD-IR or DMARD-intolerant patients, efficacy was comparable to that of tocilizumab monotherapy and combination therapy [14]. In two reports, combination therapy with MTX appeared more effective than tocilizumab monotherapy. However, one study [15] was a small dose-finding study in MTX-IR patients, and the other study [16] was a retrospective analysis of clinical practice in Japan. In the latter, results may be explained by differences in baseline characteristics, for which estimates were not adjusted; in particular, disease activity, a clear predictor of lower remission rates, was higher for monotherapy patients than for combination therapy patients [16].

Monotherapy with TNFi agents has also been studied. Results from ReACT, a study of adalimumab with a design similar to that of ACT-SURE, showed larger differences between TNFi monotherapy and combination therapy [17]. In addition, randomized controlled trials indicate that a TNFi agent plus MTX is more effective than either alone [18–20].

In summary, in this 6-month study, tocilizumab had a comparable safety profile and was similarly effective when used as monotherapy or as combination therapy with DMARDs in a broader population of patients. These data further support that tocilizumab monotherapy is a feasible treatment alternative for patients for whom combination therapy with MTX is not an option.

## Electronic supplementary material

Below is the link to the electronic supplementary material.

ESM 1 (DOCX 1216 kb)
