# Supplementary material for: Comparison of tocilizumab as monotherapy or with add-on disease-modifying antirheumatic drugs in patients with rheumatoid arthritis and inadequate responses to previous treatments: an open-label study close to clinical practice
Source: Clin Rheumatol. 2015 Jan 22;34(3):563–71. doi: 10.1007/s10067-014-2857-y (PMC4348534; doi:10.1007/s10067-014-2857-y)

**Online Resource**

**Submitted to *Clinical Rheumatology***

**Comparison of tocilizumab as monotherapy or with add-on disease-modifying antirheumatic drugs in patients with rheumatoid arthritis and inadequate responses to previous treatments: an open-label study close to clinical practice**

Vivian P. Bykerk^1,2^, Andrew J. K. Östör^3^, José Alvaro-Gracia^4^, Karel Pavelka^5^,
José Andrés Román Ivorra^6^, Winfried Graninger^7^, William Bensen^8^, Michael T. Nurmohamed^9^, Andreas Krause^10^, Corrado Bernasconi^11^, Maher Aassi^11^, Jean Sibilia^12^

^1^Inflammatory Arthritis Center, Hospital for Special Surgery, New York, New York, USA; ^2^Department of Rheumatology, Mount Sinai Hospital, Toronto, Ontario, Canada; ^3^University of Cambridge, Cambridge, United Kingdom; ^4^Hospital Universitario de la Princesa, IIS Princesa, Madrid, Spain; ^5^Institute of Rheumatology, Prague, Czech Republic; ^6^Hospital Universitario La Fe, Valencia, Spain; ^7^Medical University of Graz, Graz, Austria; ^8^St Joseph's Hospital/McMaster University, Hamilton, Ontario, Canada; ^9^VU University Medical Center, Amsterdam, Netherlands; ^10^Immanuel Hospital, Berlin, Germany; ^11^F. Hoffmann-La Roche, Basel, Switzerland; ^12^CHU Hautepierre, Strasbourg, France

**Corresponding Author**

Vivian P. Bykerk

E-mail: bykerkv@hss.edu

**SUPPLEMENTARY MATERIALS**

**Supplementary Table S1** Summary of the analysis using propensity scores

|  | **Coefficient estimate** | **Standard error** | ***p*** |
| --- | --- | --- | --- |
| **Logistic regression model for the determination of the propensity score** | | | |
| Intercept | 6.32 | 1.35 | <0.001 |
| TNFi use: prior (vs naive) | –1.02 | 0.22 | <0.001 |
| TNFi use: present (vs naive) | –1.48 | 0.19 | <0.001 |
| Comorbidities: 1 or >2 (vs 0) | -0.065 | 0.15 | 0.67 |
| Comorbidities: >2 (vs 0) | -0.14 | 0.15 | 0.34 |
| Age, years | –0.012 | 0.0072 | 0.11 |
| Sex (male) | 0.26 | 0.24 | 0.27 |
| Smoker | 0.093 | 0.20 | 0.65 |
| RA duration, years | –0.0013 | 0.0093 | 0.89 |
| Baseline Hb, g/L | –0.015 | 0.0072 | 0.036 |
| Baseline FACIT-Fatigue score | 0.032 | 0.012 | 0.0010 |
| Baseline HAQ-DI | –0.26 | 0.21 | 0.21 |
| Baseline swollen joint count | 0.0050 | 0.010 | 0.63 |
| Baseline tender joint count | –0.0035 | 0.0067 | 0.60 |
| log(Baseline CRP), mg/dL | –0.19 | 0.065 | 0.0030 |
| General health VAS, mm | 0.0015 | 0.0070 | 0.83 |
| Baseline VAS physician | –0.0025 | 0.0059 | 0.67 |
| Baseline VAS pain | –0.0026 | 0.0063 | 0.68 |
| Baseline SF-36 MCS | –0.019 | 0.0087 | 0.030 |
| Baseline SF-36 PCS | –0.031 | 0.015 | 0.047 |
| **Logistic regression results for ACR50** | | | |
| Intercept | 1.12 | 1.27 | 0.38 |
| TCZ + DMARDs (vs monotherapy) | 0.057 | 0.16 | 0.73 |
| Baseline DAS28 | 0.20 | 0.054 | <0.001 |
| TNFi use: prior (vs naive) | –0.68 | 0.20 | <0.001 |
| TNFi use: present (vs naive) | –0.47 | 0.25 | 0.059 |
| Age, years | –0.0042 | 0.0048 | 0.38 |
| Sex (male) | 0.14 | 0.14 | 0.32 |
| RA duration, years | –0.013 | 0.0066 | 0.050 |
| Baseline HAQ-DI | –0.35 | 0.11 | 0.0016 |
| Propensity score | –1.33 | 1.12 | 0.24 |
| **Linear regression results for DAS28** | | | |
| Intercept | –2.79 | 0.78 | <0.001 |
| TCZ + DMARDs (vs monotherapy) | –0.086 | 0.10 | 0.39 |
| Baseline DAS28 | –0.58 | 0.032 | <0.001 |
| TNFi use: prior (vs naive) | 0.49 | 0.12 | <0.001 |
| TNFi use: present (vs naive) | 0.76 | 0.15 | <0.001 |
| Age, years | 0.0024 | 0.0029 | 0.41 |
| Sex (male) | –0.31 | 0.085 | <0.001 |
| RA duration, years | 0.0091 | 0.0040 | 0.023 |
| Baseline HAQ-DI | 0.25 | 0.066 | <0.001 |
| Propensity score | 2.47 | 0.69 | <0.001 |

DMARD, disease-modifying antirheumatic drug; FACIT–Fatigue, Functional Assessment of Chronic Illness Therapy–Fatigue; HAQ-DI, Health Assessment Questionnaire–Disability Index; Hb, hemoglobin; RA, rheumatoid arthritis; TCZ, tocilizumab; TNFi, tumor necrosis factor-α inhibitor; VAS, Visual Analogue Scale

**Supplementary Table S2** ACR core set parameters over time

| **Week** | **Baseline** | **4** | **8** | **12** | **16** | **20** | **24** |
| --- | --- | --- | --- | --- | --- | --- | --- |
|  |  | **Tocilizumab monotherapy**  ***n*=239** | | | | | |
| SJC (66 joints) | 13.84 (10.36) | 10.23 (10.67) | 7.67 (10.14) | 6.87 (10.21) | 6.55 (10.54) | 6.11 (10.25) | 5.95 (10.77) |
| TJC (68 joints) | 24.53 (15.47) | 19.08 (16.12) | 14.78 (15.39) | 13.32 (15.80) | 12.29 (15.17) | 11.38 (14.38) | 11.09 (14.84) |
| Patient global VAS, mm | 66.55 (20.70) | 51.16 (25.04) | 38.18 (24.79) | 34.62 (24.48) | 33.48 (25.63) | 31.65 (24.52) | 28.96 (24.69) |
| Physician global VAS, mm | 63.17 (18.11) | 43.91 (21.10) | 31.98 (19.97) | 28.39 (19.59) | 25.95 (19.05) | 22.82 (18.57) | 21.80 (19.95) |
| Patient pain VAS, mm | 61.26 (22.45) | 47.24 (24.45) | 35.68 (24.72) | 32.28 (24.19) | 30.95 (24.89) | 28.94 (23.17) | 27.24 (23.53) |
| CRP, mg/dL | 2.32 (2.79) | 0.67 (1.68) | 0.42 (1.24) | 0.30 (0.79) | 0.26 (0.73) | 0.25 (0.82) | 0.31 (0.94) |
| ESR, mm/h | 42.50 (28.06) | 15.56 (19.01) | 11.90 (15.47) | 9.98 (13.20) | 9.62 (15.39) | 8.77 (15.13) | 10.00 (16.31) |
| HAQ-DI | 1.68 (0.63) | 1.48 (0.69) | 1.30 (0.69) | 1.19 (0.72) | 1.17 (0.72) | 1.16 (0.74) | 1.15 (0.77) |
|  |  | **Tocilizumab + DMARD(s)**  ***n*=1442** | | | | | |
| SJC (66 joints) | 12.29 (8.67) | 9.07 (10.32) | 6.37 (8.51) | 5.41 (7.95) | 5.02 (8.03) | 4.54 (7.73) | 4.37 (7.69) |
| TJC (68 joints) | 21.46 (14.35) | 16.13 (14.80) | 11.79 (12.79) | 10.00 (12.21) | 9.34 (11.80) | 8.97 (11.92) | 8.35 (11.56) |
| Patient global VAS, mm | 61.85 (21.20) | 46.54 (23.70) | 37.92 (24.32) | 33.05 (23.31) | 30.50 (23.38) | 28.26 (22.65) | 26.29 (21.59) |
| Physician global VAS, mm | 58.91 (17.61) | 39.11 (19.57) | 29.69 (18.44) | 25.27 (17.95) | 22.72 (17.38) | 20.91 (16.80) | 19.15 (16.65) |
| Patient pain VAS, mm | 56.89 (22.55) | 42.42 (23.35) | 34.81 (23.55) | 30.82 (23.20) | 28.17 (22.68) | 26.41 (22.33) | 24.51 (21.00) |
| CRP, mg/dL | 1.78 (2.71) | 0.48 (1.83) | 0.34 (1.37) | 0.28 (1.30) | 0.23 (0.82) | 0.23 (1.11) | 0.17 (0.77) |
| ESR, mm/h | 37.83 (26.55) | 12.69 (15.90) | 10.05 (14.81) | 8.76 (12.46) | 8.21 (11.87) | 7.61 (10.90) | 7.65 (10.63) |
| HAQ-DI | 1.46 (0.63) | 1.24 (0.65) | 1.09 (0.67) | 1.01 (0.68) | 0.96 (0.68) | 0.92 (0.67) | 0.89 (0.68) |

CRP, C-reactive protein; DMARD, disease-modifying antirheumatic drug; ESR, erythrocyte sedimentation rate; HAQ-DI, Health Assessment Questionnaire–Disability Index; SJC, swollen joint count; TJC, tender joint count; VAS, Visual Analogue Scale

Data are presented as mean (SD). Not all data are available for the full sample (239 and 1442 patients in the two respective groups) at all time points. Imputation (last-observation-carried-forward up to withdrawal) was performed for joint counts only

**Supplementary Table S3** Comparison of week 24 efficacy outcomes between tocilizumab monotherapy and combination DMARD therapy in subsets of TNF-IR and DMARD-IR patients

|  | **DMARD-IR** | |  | **TNFi-IR** | |  |
| --- | --- | --- | --- | --- | --- | --- |
|  | **Tocilizumab monotherapy**  ***N*=66** | **Tocilizumab + DMARD(s)**  ***N*=910** | ***p* for difference^a^** | **Tocilizumab monotherapy**  ***N*=173** | **Tocilizumab + DMARD(s)**  ***N*=532** | ***p* for difference^a^** |
| ACR20 responders, *n* (%) | 48 (72.7) | 640 (70.3) | 0.6985 | 112 (64.7) | 324 (60.9) | 0.6016 |
| ACR50 responders, *n* (%) | 32 (48.5) | 475 (52.2) | 0.5498 | 72 (41.6) | 205 (38.5) | 0.7803 |
| ACR70 responders, *n* (%) | 18 (27.3) | 292 (32.1) | 0.4046 | 39 (22.5) | 94 (17.7) | 0.2153 |
| DAS28 change from baseline, mean ± SD | –3.57 **±** 1.434  *n*=63 | –3.52 **±** 1.362  *n*=802 | 0.8800 | –3.34 **±** 1.514  *n*=142 | –3.26 **±** 1.531  *n*=444 | 0.4302 |
| CDAI change from baseline, mean ± SD | –23.90 **±** 13.824  *n*=64 | –24.05 **±** 13.018  *n*=805 | 0.9224 | –23.90 **±** 14.860  *n*=144 | –22.70 **±** 14.060  *n*=433 | 0.4002 |
| SDAI change from baseline, mean ± SD | –26.06 **±** 14.975  *n*=63 | –25.63 **±** 13.507  *n*=786 | 0.9358 | –26.09 **±** 15.399  *n*=140 | –24.55 **±** 14.936  *n*=427 | 0.4081 |

CDAI, clinical disease activity index; DAS28, disease activity score based on 28 joints; DMARD, disease-modifying antirheumatic drug; IR, inadequate response; SDAI, simplified disease activity index; TNF, tumor necrosis factor; TNFi, tumor necrosis factor-α inhibitor

*N*=number of patients with assessments

^a^Logistic regression model used for comparison of ACR20/50/70 response and linear regression model used for comparison of DAS28, CDAI and SDAI change from baseline

**Supplementary Table S4** Key baseline demographics, baseline disease characteristics, and effectiveness outcomes in patients who received methotrexate

| **Baseline demographics and disease characteristics** | **Patients on MTX at baseline**  ***n*=1134** |
| --- | --- |
| Age, years | 52.9 (12.5) |
| Female, n (%) | 905 (79.8) |
| Duration of RA, years | 9.0 (8.5) |
| TNFi previous use, n (%)^a^ | 190 (16.8) |
| TNFi recent use, n (%)^b^ | 227 (20.0) |
| DAS28 at baseline | 5.93 (1.17) |
| **Effectiveness at week 24** | |
| DAS28 | 2.51 (1.36) |
| Change in DAS28 from baseline to week 24 | –3.39 (1.43) |
| ACR responders, n (%) |  |
| ACR20 | 759 (66.9) |
| ACR50 | 531 (46.8) |
| ACR70 | 293 (25.8) |
| EULAR good/moderate responders at week 24, n (%) | 956 (84.3) |

ACR, American College of Rheumatology; DAS28, disease activity score based on 28 joints; EULAR, European League Against Rheumatism; MTX, methotrexate; RA, rheumatoid arthritis; TNFi, tumor necrosis factor-α inhibitor

Data are mean (standard deviation) unless stated otherwise

^a^Patients who did not use TNFi for >2 months before baseline

^b^Patients who used TNFi for ≤2 months before baseline

**Supplementary Table S5** Effectiveness of tocilizumab during the first 6 months of the extension study (extension study population, *N*=934)

|  | ***n*** | **TCZ monotherapy** | ***n*** | **TCZ + DMARD(s)** |
| --- | --- | --- | --- | --- |
| DAS28 | | | | |
| Mean |  |  |  |  |
| Week 12 | 107 | 2.49 | 784 | 2.38 |
| Week 24 | 85 | 2.53 | 674 | 2.33 |
| Change from baseline | | | | |
| Week 12 | 107 | –3.63 | 782 | –3.58 |
| Week 24 | 85 | –3.60 | 672 | –3.62 |
| Remission (<2.6), n (%) | | | | |
| Week 12 | 107 | 66 (61.7) | 784 | 465 (59.3) |
| Week 24 | 85 | 53 (62.4) | 674 | 417 (61.9) |
| ACR20, n (%) | | | | |
| Week 12 | 117 | 82 (70.1) | 817 | 629 (77.0) |
| Week 24 | 98 | 69 (70.4) | 729 | 552 (75.7) |
| ACR50, n (%) | | | | |
| Week 12 | 117 | 64 (54.7) | 817 | 462 (56.5) |
| Week 24 | 98 | 47 (48.0) | 729 | 399 (54.7) |
| ACR70, n (%) | | | | |
| Week 12 | 117 | 41 (35.0) | 817 | 252 (30.8) |
| Week 24 | 98 | 31 (31.6) | 729 | 225 (30.9) |
| ACR90, n (%) | | | | |
| Week 12 | 117 | 13 (11.1) | 817 | 79 (9.7) |
| Week 24 | 98 | 11 (11.2) | 729 | 76 (10.4) |
| HAQ-DI improvement ≥0.22, n (%) | | | | |
| Week 12 | 108 | 76 (70.4) | 787 | 583 (74.1) |
| Week 24 | 86 | 66 (76.7) | 679 | 499 (73.5) |

For ACR responses and HAQ-DI improvement, baseline was the first visit of the ACT-SURE study.

In 169 centers (of 264 centers participating in the ACT-SURE study), patients who completed week 24 of the main study and who experienced at least moderate EULAR response could enter a separate long-term extension study at the discretion of the investigator.

**Supplementary Fig. S1** Summary of patient disposition


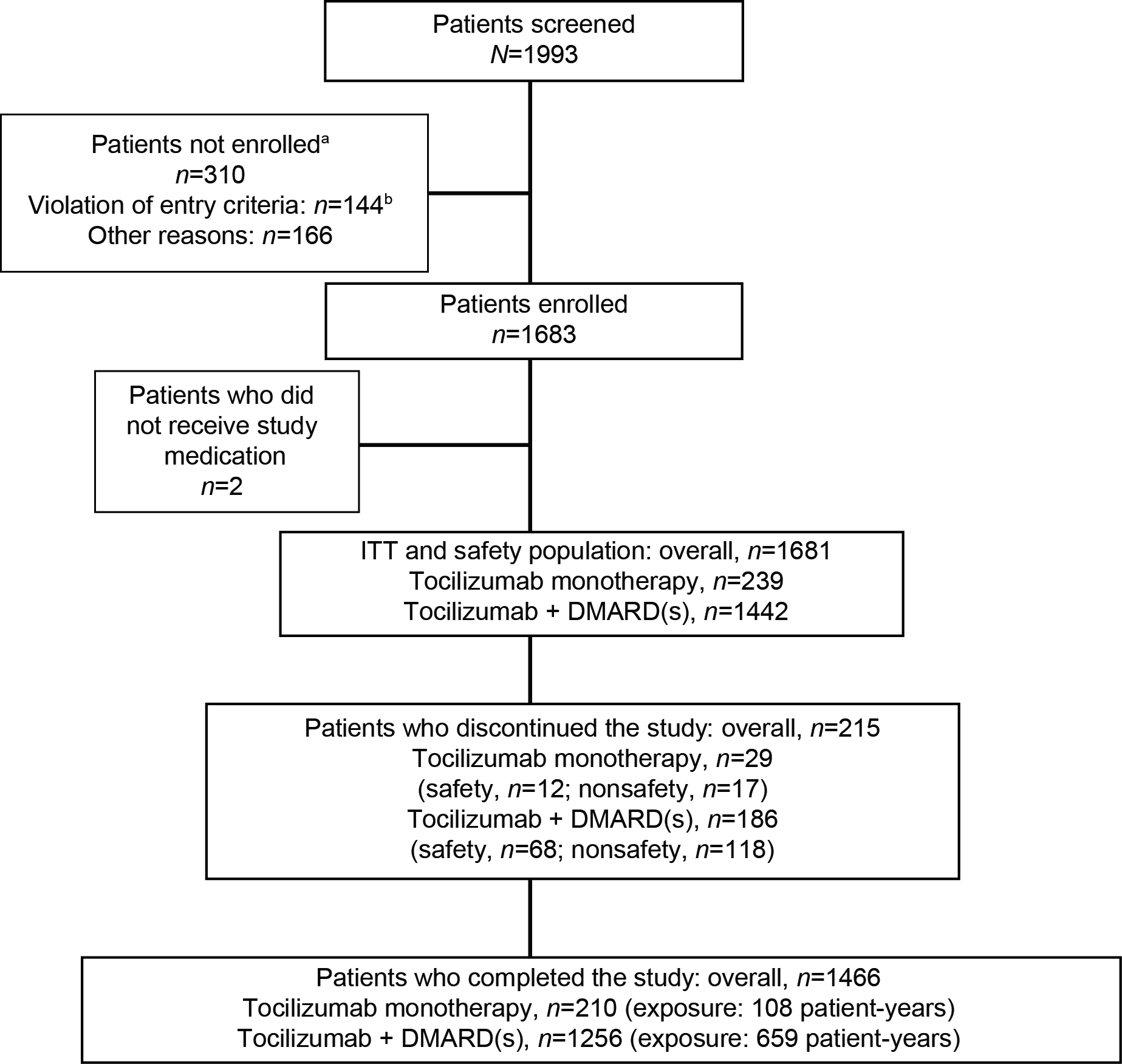


^a^Patients not enrolled: mean age 54.8 years, RA duration 9.3 years, tender joint count 20.2, swollen joint count 11.3

^b^Most common criteria leading to exclusion (each contributing to >5% of exclusions):

- Chest x-ray evidence of any clinically significant abnormality (9%)
- Patients to be screened for latent tuberculosis (TB) before biologics use, in accordance with local guidelines or Good Clinical Practice in each country. If screening results are positive, patients with latent TB should be treated with standard antimycobacterial therapy (at least 4 weeks) before initiation of tocilizumab and should have negative findings on chest x-ray for active TB at screening (8%)
- Active TB requiring treatment within the previous 3 years (7%)
- Known, active current or history of recurrent bacterial, viral, fungal, mycobacterial or other infection (including, but not limited to, TB and atypical mycobacterial disease, clinically significant abnormalities on chest x-ray as determined by the investigator, hepatitis B and C and herpes zoster, excluding fungal infections of nail beds), or any major episode of infection requiring hospitalization or treatment with intravenous antibiotics within 4 weeks of screening or with oral antibiotics within 2 weeks of screening (7%)

**Supplementary Fig. S2** Percentages of patients who achieved ACR20/50/70/90 responses over time. Nonresponder imputation for patients who withdrew or for whom responses were missing. *p* values were calculated by logistic regression analysis adjusted for previous treatment (DMARD-IR/TNFi-IR prior use/TNF-IR recent use) and baseline DAS28. DMARD, disease-modifying antirheumatic drug; IR, inadequate response; TCZ, tocilizumab; TNFi, tumor necrosis factor-α inhibitor


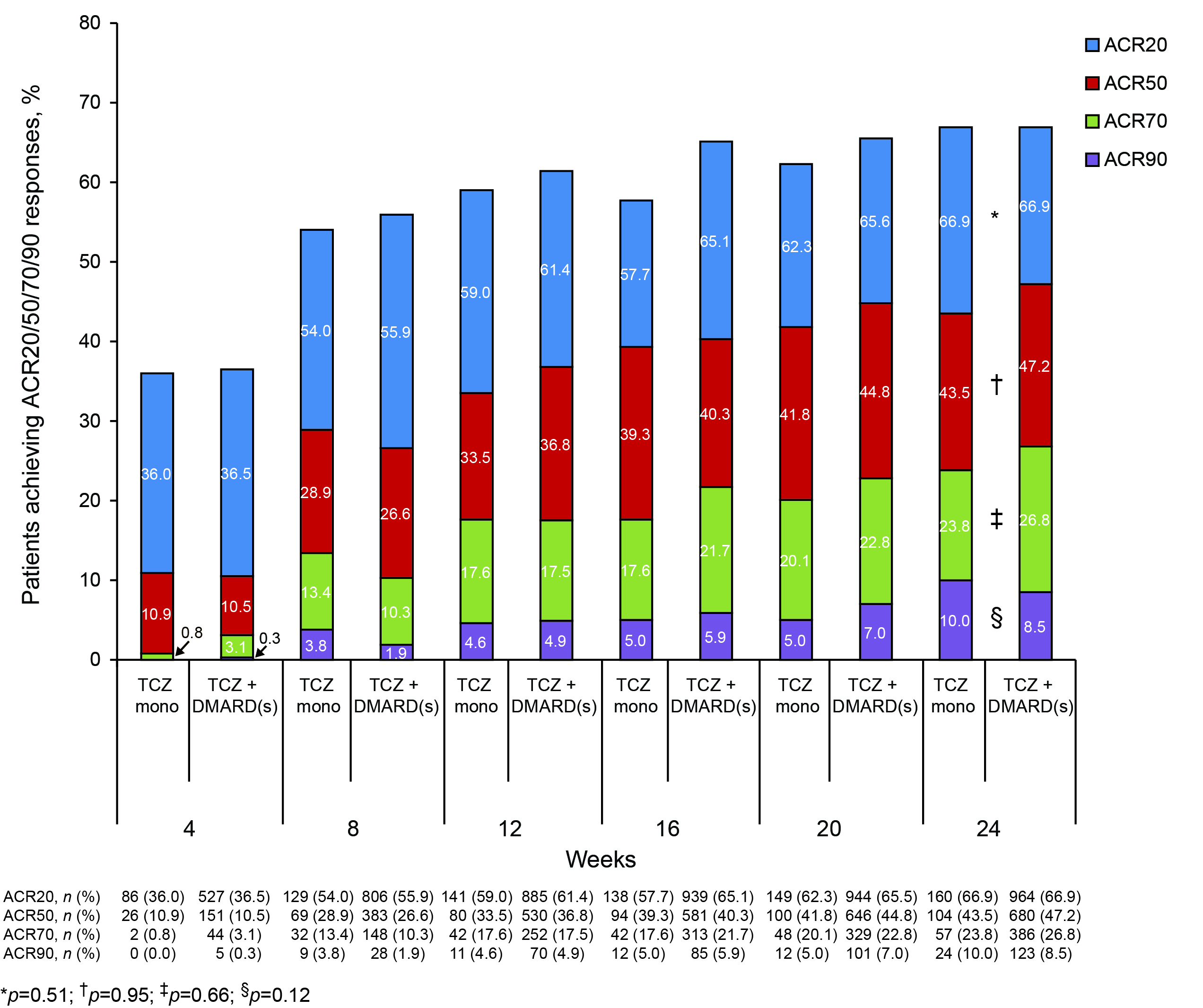


**Supplementary Fig. S3** Proportions of patients who achieved efficacy responses over time. (**a**) EULAR good and/or moderate responses. (**b**) DAS28 <2.6. (**a**) Nonresponder imputation for patients who withdrew or for whom responses were missing. Missing data for joint counts only were imputed using the last-observation-carried-forward approach up to withdrawal. (**b**) Patients for whom data were missing were not included. *p* values calculated by logistic regression analysis adjusted for previous treatment (DMARD-IR/TNFi-IR prior use/TNF-IR recent use) and baseline DAS28. DMARD, disease-modifying antirheumatic drug; IR, inadequate response; TCZ, tocilizumab; TNFi, tumor necrosis factor-α inhibitor


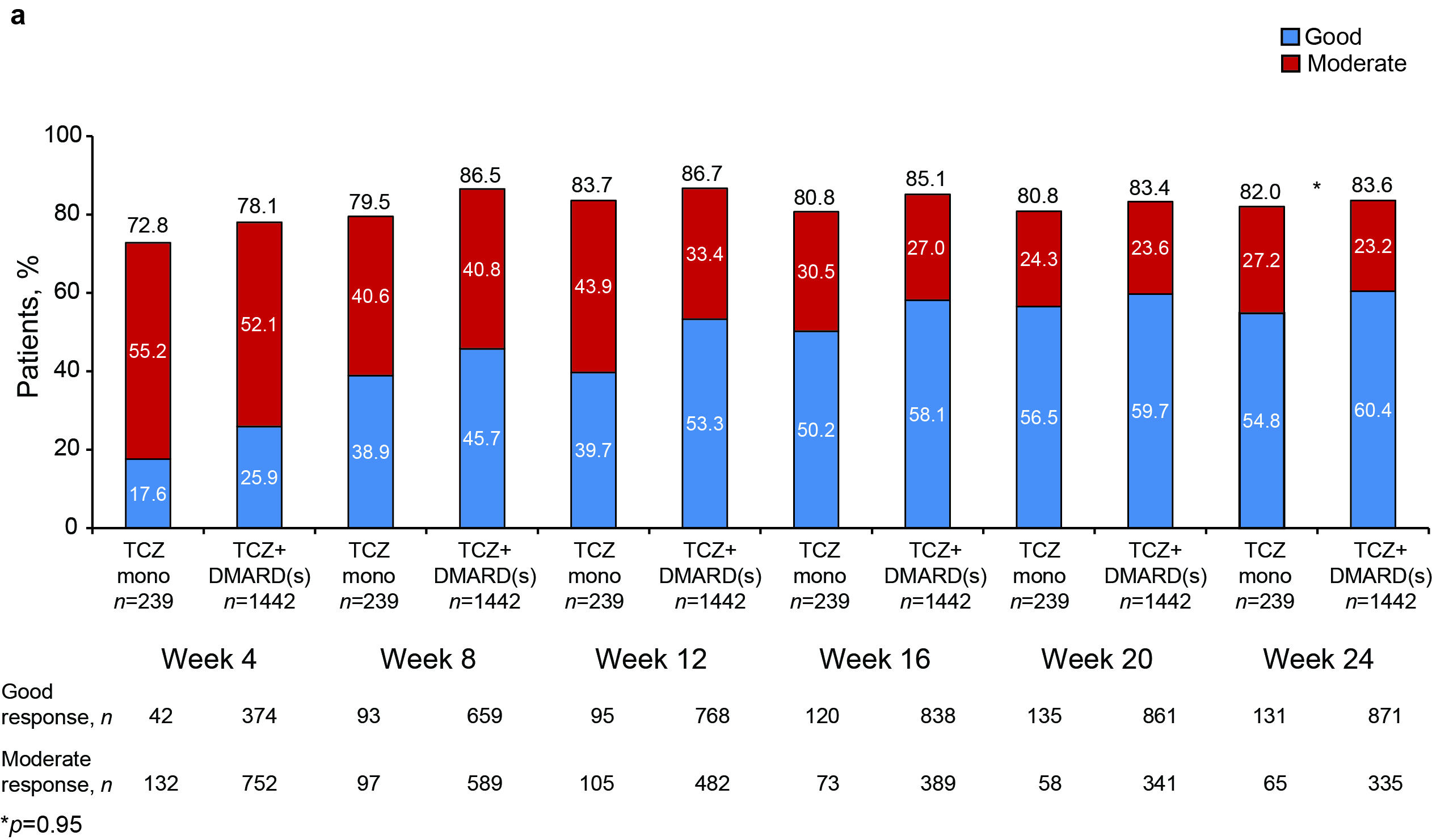


**
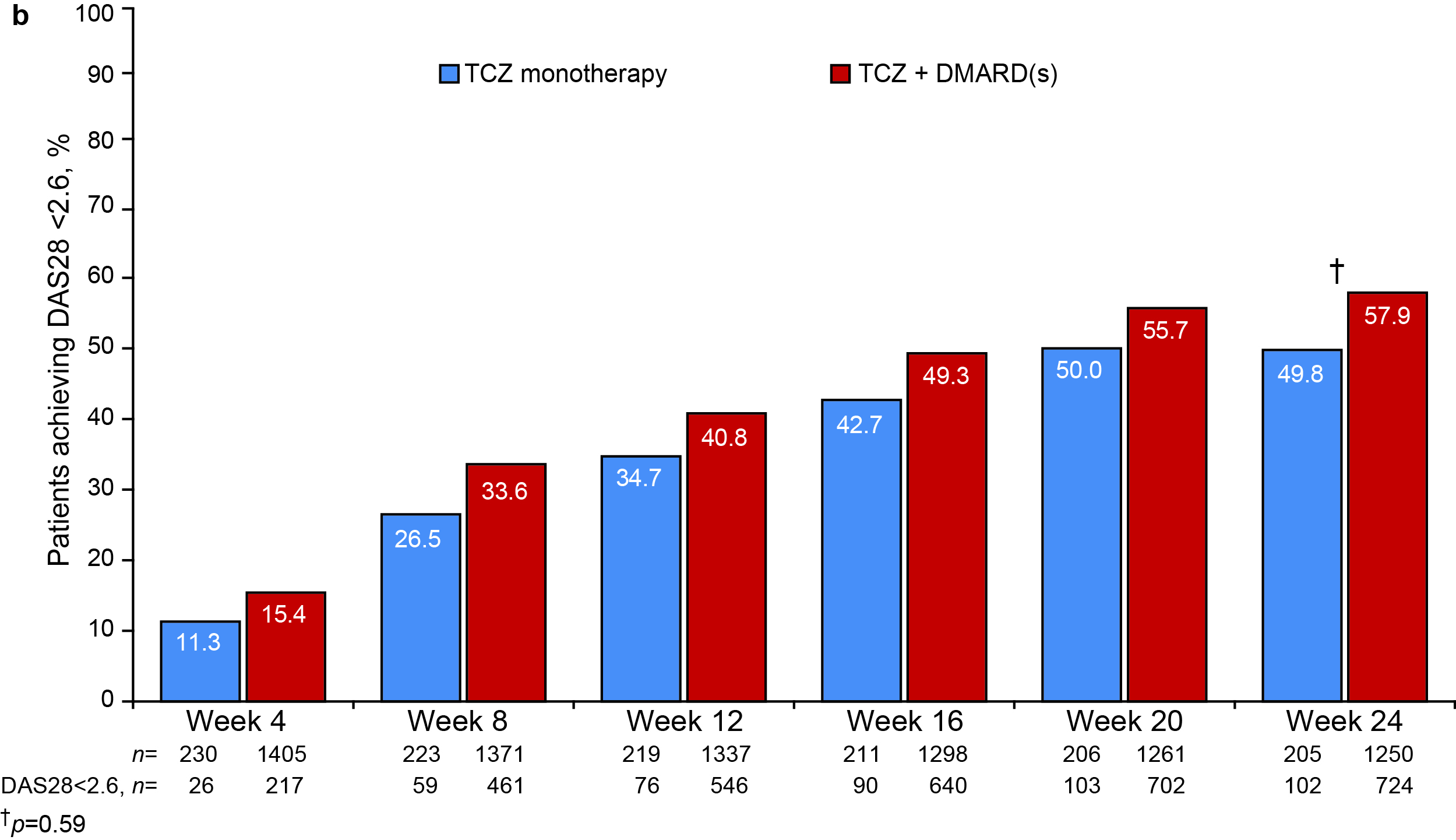
**

**Supplementary Fig. S4** Percentages of patients who achieved ≥0.22 HAQ-DI reduction from baseline over time. The *p* value was adjusted for previous treatment (DMARD-IR/TNFi-IR prior use/TNF-IR recent use), baseline DAS28, and baseline HAQ-DI. Patients for whom data were missing were not included. DMARD, disease-modifying antirheumatic drug; HAQ-DI, Health Assessment Questionnaire–Disability Index; IR, inadequate response; TCZ, tocilizumab; TNFi, tumor necrosis factor-α inhibitor


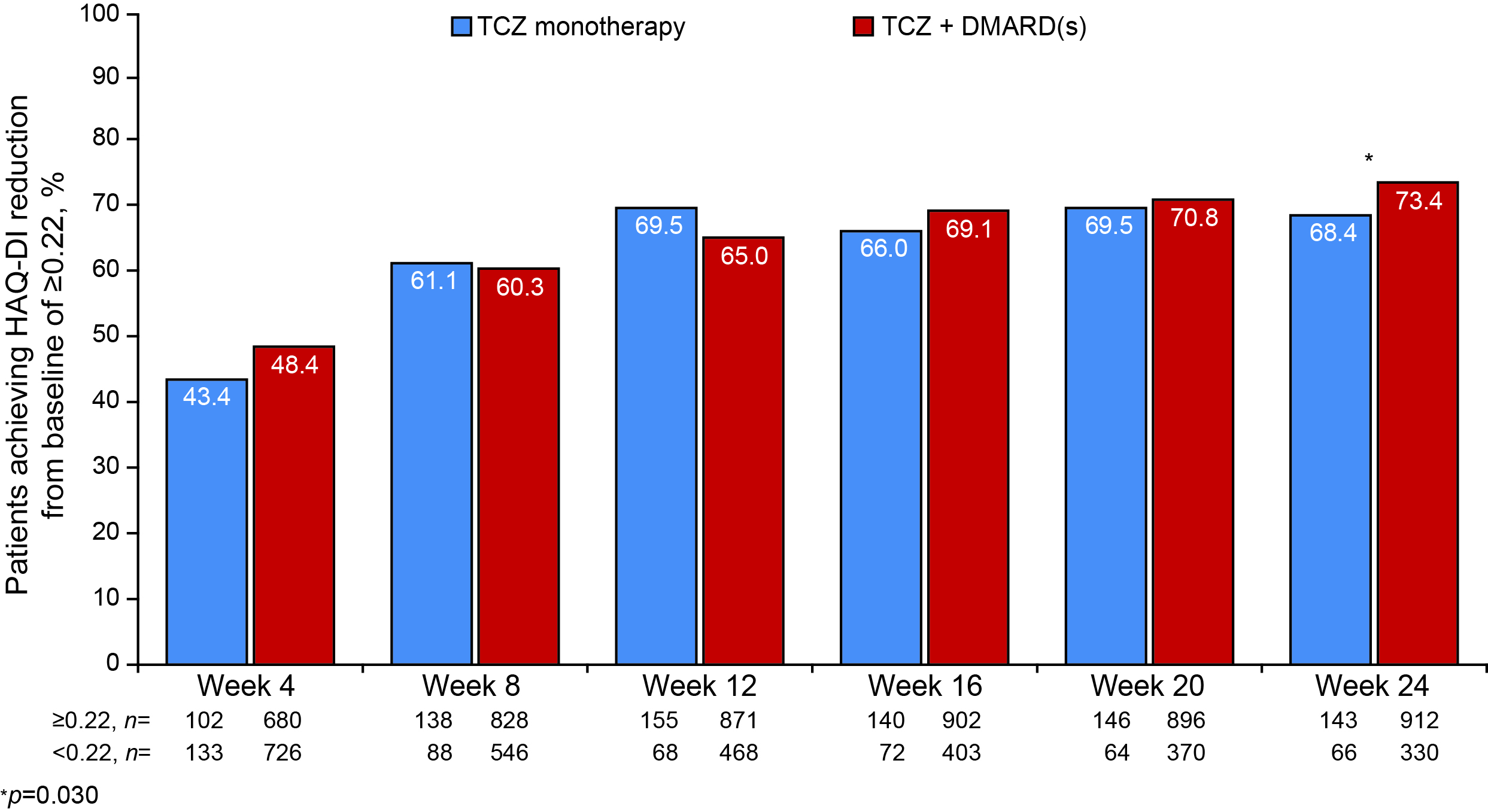

Supplement: Supplementary file 1 — (DOCX 1216 kb) [file 10067_2014_2857_MOESM1_ESM.docx]
